# The Multidimensional Dynamic Feedback Model: A Comprehensive Framework for Understanding and Managing Attention-Deficit/Hyperactivity Disorder

**DOI:** 10.3390/children12030303

**Published:** 2025-02-27

**Authors:** Yuying Wang, Yuan Zhao, Luming Hu, Xuemin Zhang

**Affiliations:** 1 Beijing Key Laboratory of Applied Experimental Psychology, National Demonstration Center for Experimental Psychology Education, Faculty of Psychology, Beijing Normal University, Beijing 100875, China; 2 Faculty of Psychology and Educational Sciences, KU Leuven, 3000 Leuven, Belgium; 3 Department of Psychology, School of Arts and Sciences, Beijing Normal University at Zhuhai, Zhuhai 519085, China; 4 State Key Laboratory of Cognitive Neuroscience and Learning, Beijing Normal University, Beijing 100875, China

**Keywords:** ADHD, pediatric health, behavioral feedback loops, personalized interventions, prevention and management

## Abstract

Attention-deficit/hyperactivity disorder (ADHD) is a common neurodevelopmental disorder characterized by persistent inattention, hyperactivity, and impulsivity. These symptoms can significantly impact academic performance, social interactions, and daily activities, often creating cycles that worsen long-term challenges. This review introduces the *Multidimensional Dynamic Feedback Model* (MDFM), which aids in understanding ADHD’s development and guiding intervention strategies. The model emphasizes the dynamic interactions among genetic, environmental, cognitive, and behavioral factors. The MDFM consists of three key aspects: (1) the interplay between genetic and environmental factors in shaping ADHD’s biological basis, (2) the role of cognitive and neural processes in driving core symptoms, and (3) the influence of behavioral feedback loops that reinforce negative behaviors and hinder adaptation. The model highlights the importance of personalized interventions and effective feedback systems, including early prevention, supportive family and school environments, and the impact of social and cultural backgrounds on treatment outcomes. As a comprehensive framework, the MDFM offers a holistic perspective for clinicians, aiming to enhance long-term outcomes and promote the health and well-being of individuals with ADHD across the lifespan. By addressing implementation challenges, the model seeks to improve ADHD prevention and management, ultimately supporting individuals and their communities.

## 1. Introduction

Attention-deficit/hyperactivity disorder (ADHD) is a neurodevelopmental disorder that affects about 8% of children and adolescents worldwide [1,2]. It is defined by ongoing problems with inattention, hyperactivity, and impulsivity. These symptoms are linked to difficulties in working memory, cognitive flexibility, inhibitory control, and emotional regulation [3,4]. ADHD often appears early in childhood and can lead to academic struggles, emotional distress, and a higher risk of accidents and injuries [5,6,7,8]. Without proper intervention, these issues often continue into adolescence and adulthood, causing long-term problems such as social isolation, lower quality of life, and more health complications [9,10,11]. ADHD also creates challenges for families, schools, and society. Parents and caregivers often experience high levels of stress, and teachers face difficulties in meeting the needs of children with ADHD in the classroom [12,13,14]. These issues highlight the need to better understand ADHD’s causes and develop effective, evidence-based treatments.

Over the past several decades, research has shown that ADHD results from a complex interaction of genetic, environmental, and neurocognitive factors. Environmental factors, such as prenatal stress, maternal smoking, and early family environments, significantly increase the risk of ADHD, especially during critical periods of brain development [15,16,17,18,19]. Genetic predispositions also play a critical role, with studies indicating that first-degree relatives of individuals with ADHD are two to eight times more likely to develop the disorder [20,21]. Epigenetic mechanisms further connect genetic and environmental factors by regulating gene expression in response to influences like prenatal stress and nutrition [22,23]. At the neurocognitive level, ADHD is linked to imbalances in neurotransmitters such as dopamine, norepinephrine, and serotonin. It is also associated with structural and functional abnormalities in brain regions involved in executive functions, including the prefrontal cortex and caudate nucleus [24,25,26,27,28,29]. Building on these findings, theoretical models have been developed to explain ADHD. The Executive Function Model suggests that the core problem lies in deficits in executive functions, such as working memory, self-regulation, inhibitory control, and time management. These deficits lead to the key symptoms of inattention, impulsivity, and hyperactivity, which are tied to disruptions in prefrontal inhibitory control [30]. The Dual Pathway Model proposes two main pathways: one related to executive dysfunction and the other to motivational deficits, which are reflected in a heightened aversion to delayed rewards. This model helps explain the social and academic difficulties often seen in children with ADHD [31,32].

While these models have provided critical insights into ADHD’s etiology, much of the current research remains fragmented, often isolating environmental, genetic, or neurocognitive factors. These reductionist approaches overlook the multifactorial nature of ADHD, limiting our understanding of its development and constraining the creation of holistic interventions. Recent studies on heterogeneity and multiple causal pathways suggest that ADHD arises from the interaction of various deficits, including problems with executive functioning, reward and motivation anomalies, and difficulties in emotional regulation [33]. This perspective aligns with the widely recognized Bio-Psycho-Social Model (BPSM) [34,35], which emphasizes the importance of considering biological, psychological, and social factors together [35]. By considering these factors together, the BPSM provides a more comprehensive view than fragmented approaches and serves as a key framework for guiding clinical decisions and designing effective interventions [36]. However, the BPSM and similar integrative models have a significant limitation: they adopt a static perspective and fail to account for the dynamic interplay of biological, psychological, and environmental factors over time. These models do not fully capture how these influences evolve, interact, and shape the developmental trajectory of ADHD.

To address these limitations, this review introduces a multidimensional dynamic model for ADHD. This model integrates environmental influences, genetic predispositions, neurocognitive mechanisms, and behavioral patterns within a dynamic framework. By highlighting the reciprocal interactions and feedback loops among these factors, the MDFM provides a more comprehensive understanding of ADHD as a multidimensional neurodevelopmental disorder. It also lays the groundwork for developing personalized, multi-level interventions tailored to the unique needs of affected populations, particularly children and adolescents, aiming to improve long-term outcomes and quality of life.

## 2. The Multidimensional Dynamic Feedback Model for ADHD

The *Multidimensional Dynamic Feedback Model* (MDFM) offers a comprehensive framework for understanding ADHD as a dynamic and interconnected process. Unlike static models that focus on isolated factors, the MDFM emphasizes the reciprocal and cyclical interactions among genetic predispositions, environmental influences, neurocognitive mechanisms, and behavioral patterns. By viewing ADHD as a multidimensional system, the model incorporates feedback loops and dynamic interactions across multiple levels, offering a holistic perspective on ADHD.

**Figure 1:**
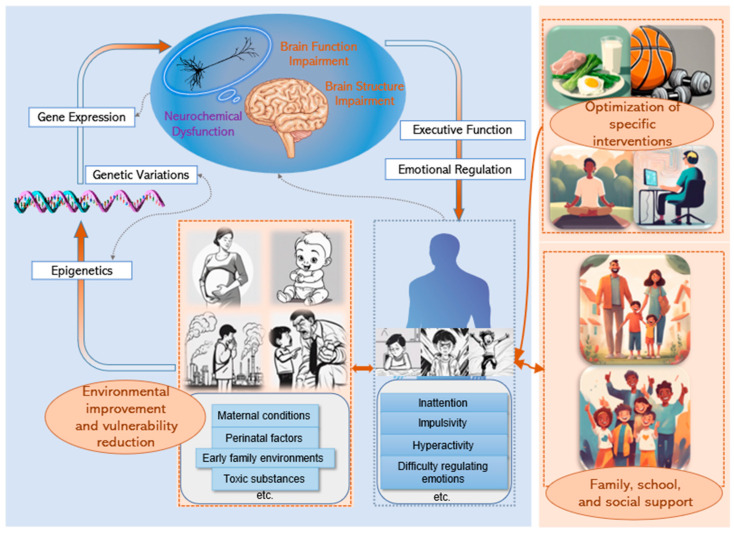
The Multidimensional Dynamic Feedback Model for ADHD and the Multidimensional Dynamic Approaches to ADHD Intervention. **The blue-shaded area shows the *Multidimensional Dynamic Feedback Model***. Following the direction of the arrows, this model begins by illustrating how prenatal and early postnatal genetic factors, epigenetics, and environmental influences impact gene expression. These factors result in dysregulation of neurotransmitters, as well as impairments in brain function and structure. Consequently, individuals may have difficulties with executive functioning and emotional regulation. These issues can show up as inattention, impulsivity, hyperactivity, and emotional problems. Such behaviors can lead to negative feedback from family, school, and society, creating a cycle that worsens ADHD symptoms. **The orange-shaded area represents multidimensional dynamic approaches to ADHD intervention.** In early life, interventions focus on prevention through good nutrition, maternal health, and reducing exposure to harmful substances. For children and adolescents, personalized interventions combine traditional methods with new techniques, along with support from family, school, and community. For adults with ADHD, similar strategies should be implemented, with adjustments made to address their specific needs in social situations. Clinical professionals are essential for making intervention decisions in this system. This figure includes original elements created by the author and incorporates additional elements from Bioicons (https://bioicons.com, accessed on 10 January 2025), including “Patient” by Marcel Tisch, “Pyramidal Neuron” by Fabian Mikulasch, “DNA Symbolic Extending” by David Eccles (Gringer) (all under CC0), and “Brain-2” by Servier (licensed under CC BY 3.0 Unported).

The MDFM is built around three interconnected dimensions: the interplay of genetic and environmental factors, the neurocognitive processes linking biology and behavior, and the regulatory feedback loops that sustain behavioral symptoms. These dimensions form an evolving system, highlighting the importance of temporal dynamics in shaping ADHD trajectories (as highlighted in the blue-shaded area of Figure 1). By capturing these processes, the MDFM deepens our understanding of ADHD and provides a strong foundation for designing adaptive, multi-level interventions tailored to the unique and changing needs of individuals.

### 2.1. Fundamental Interplay in ADHD: The Role of Environment and Genetics

The first dimension of the model demonstrates how environmental and genetic factors interact to shape biological susceptibility to ADHD. Research has shown that ADHD arises from a combination of genetic predispositions and environmental influences, which interact through complex, dynamic mechanisms [37,38]. Adverse prenatal conditions, such as maternal stress, poor nutrition, and toxin exposure, can alter gene expression through epigenetic mechanisms like DNA methylation [37,39,40]. These modifications affect key neurotransmitter systems, such as dopamine and norepinephrine, which are critical to neurodevelopmental pathways linked to ADHD. For example, methylation of dopamine receptor genes (e.g., DRD4, DRD5) has been associated with increased ADHD risk, underscoring the role of epigenetic regulation in shaping susceptibility [41,42,43]. In parallel, polygenic variations in genes related to neurotransmitter pathways contribute to ADHD risk by disrupting neural circuits involved in attention, impulse control, and emotional regulation [27,44]. Environmental factors, such as prenatal stress and nutritional deficiencies, can amplify these genetic vulnerabilities through epigenetic modifications, creating a dynamic interplay between inherited predispositions and external influences [45,46].

This interaction between genetics and environment emphasizes the need for longitudinal studies to explore how these factors evolve over time to influence ADHD risk. Such research could use genetic testing, epigenetic profiling, and environmental monitoring to identify critical periods of vulnerability and resilience. Clinicians could apply these insights to implement early preventive measures, such as reducing prenatal risk factors or providing nutritional support during key developmental stages.

### 2.2. Neurocognitive Interactions in ADHD: Bridging Biological and Behavior

The second dimension of the model focuses on neurocognitive mechanisms, which act as intermediaries between genetic and environmental influences and the behavioral symptoms of ADHD. These mechanisms translate molecular disruptions into neural dysfunctions that underlie ADHD’s core symptoms.

At the molecular level, dysregulation of neurotransmitters like dopamine and norepinephrine impairs executive functioning and behavioral inhibition, contributing to inattention and impulsivity [26,27]. These disruptions affect broader neural networks, particularly the cortico-striato-thalamo-cortical (CSTC) circuit, which is critical for cognitive control and impulsivity regulation [47]. ADHD is also associated with structural and functional abnormalities in key brain regions, such as the prefrontal cortex, caudate nucleus, and hippocampus, especially during childhood [26,29,48,49,50]. Neuroimaging studies show reduced gray matter volume and decreased activation in frontal regions responsible for executive functions, as well as reduced connectivity within key brain networks, including the default mode network (DMN) and circuits governing attention and cognitive control. These abnormalities are directly linked to ADHD’s hallmark symptoms of inattention and impulsivity [28,51,52].

To further validate this neurocognitive dimension, future research could integrate genetic data, neuroimaging findings, and behavioral assessments into longitudinal studies. However, since ADHD-related neural differences are often detectable at the cohort level rather than the individual level [50], large-scale collaborations or open datasets (e.g., ENIGMA-ADHD) are essential to ensure sufficient sample sizes and statistical power [53]. Despite the challenges, identifying subtle ADHD-specific alterations in neural connectivity (such as slight changes in neurotransmitter levels) is crucial. These alterations, particularly within the CSTC circuit, could provide critical insights. They may help distinguish ADHD from other neurodevelopmental disorders [47,54]. Such findings, alongside advancements in technology, could, in the future, inform more precise diagnoses and guide targeted interventions.

### 2.3. Regulatory Interactions in ADHD: Feedback Mechanisms in Cognition and Behavior

The third dimension of the model underscores the regulatory feedback loops that link the behavioral patterns of ADHD with underlying cognitive and neural systems. Children with ADHD often face challenges in attention and executive functioning, which can hinder their ability to complete tasks or meet expectations in regulated environments. These struggles frequently lead to negative feedback from teachers or peers, contributing to frustration, a loss of confidence, and heightened stress as children cope with repeated failures [55,56,57]. Prolonged stress can result in persistently high cortisol levels, disrupting the prefrontal cortex’s role in regulating attention and executive functioning. This physiological response destabilizes neural networks involved in executive functioning, further worsening ADHD symptoms and impairing cognitive flexibility [58,59]. To illustrate this cycle, consider children who struggle with math assignments. During a lesson, some children find it difficult to focus and follow the teacher’s instructions. As a result, they complete only part of the assignment or make many errors. The teacher provides critical feedback, highlighting incomplete or incorrect work. This negative response triggers feelings of frustration and inadequacy in children. They begin to doubt their abilities and become increasingly anxious about future assignments. This anxiety and stress further impair their ability to concentrate, creating a self-reinforcing loop that makes learning progressively more challenging. Thus, ADHD symptoms arise from neural and cognitive disruptions but also contribute to broader challenges in learning, daily functioning, and mental well-being. These challenges, in turn, amplify the underlying issues, creating a reinforcing cycle that perpetuates the condition.

Breaking this cycle requires interventions that target these feedback mechanisms. Traditional approaches, such as cognitive–behavioral therapy (CBT) and physical activity, have shown promise in improving executive functioning and emotional regulation [60,61]. Additionally, neurotechnologies such as neurofeedback and repetitive transcranial magnetic stimulation (rTMS) provide methods to modulate neural activity and promote cognitive stability [60,62,63]. For example, neurofeedback can provide objective feedback on brain activity, enabling patients to self-regulate neural responses and disrupt maladaptive feedback loops [64,65]. A meta-analysis highlighted the efficacy of neurofeedback in treating ADHD, demonstrating significant reductions in inattention, impulsivity, and hyperactivity [66]. Therefore, incorporating neurophysiological feedback technologies alongside established therapeutic approaches allows clinicians to develop a comprehensive treatment strategy. This integrated approach can effectively target the underlying neural mechanisms associated with ADHD, ultimately enhancing the likelihood of achieving improved clinical outcomes for patients.

Future research should focus on integrating multimodal approaches, such as neuroimaging, neurophysiological measures, behavioral data, and other diagnostic tools. This broader integration can enhance our understanding of how feedback-based mechanisms influence neural networks and cognitive processes. Although neuroimaging and neurophysiological techniques provide valuable insights into brain activity and network dynamics, they are often costly, hard to access, and may not accurately reflect real-life behaviors due to their controlled laboratory settings [67]. To overcome these limitations, we should develop low-cost and scalable feedback tools, including mobile applications and wearable devices. Although the monitoring data from these tools present analytical challenges, their integration with machine learning algorithms and key ADHD metrics holds significant potential for future advancements in understanding and treating ADHD [68,69]. Additionally, incorporating multimodal approaches into longitudinal studies can significantly enhance our understanding of the long-term effects of ADHD. This integration enables a more nuanced analysis of the mechanisms underlying ADHD, facilitating the refinement of treatment strategies that address its dynamic and multifaceted nature [67,70].

### 2.4. Toward a Systemic and Dynamic Understanding of ADHD

MDFM provides a comprehensive, integrative, and dynamic framework for understanding ADHD. By integrating insights from genetics, neurocognition, behavior, and environment, the model highlights the interconnectedness of biological, cognitive, and behavioral processes. Its strength lies in its ability to conceptualize ADHD as a dynamic system shaped by reciprocal interactions and feedback loops rather than static, isolated factors. While the model is grounded in extensive prior research, it advances the field by providing a unified perspective that underscores the importance of integrating multiple dimensions to grasp the complexity of ADHD. Future research should focus on validating this model through longitudinal studies and exploring its application across diverse cultural and socioeconomic contexts.

## 3. Multidimensional Dynamic Approaches to ADHD Intervention

MDFM presents a comprehensive framework for ADHD intervention by integrating environmental, genetic, cognitive–neural, and behavioral feedback mechanisms into a unified system. This model emphasizes dynamic feedback processes, which makes interventions personalized and adaptable over time. Interventions are guided by ongoing monitoring of neurophysiological markers, behavioral patterns, treatment responses, and symptom progression. This integration allows clinicians to make timely adjustments to treatment plans, optimizing both symptom management and long-term social adaptation.

The framework advocates for a holistic, individualized approach that combines foundational therapeutic strategies with cutting-edge technologies while addressing cultural, social, and resource-based disparities. The following sections will expand on this multidimensional framework through three interconnected aspects: personalized intervention optimization, systemic feedback architecture, and dynamic adaptation processes.

### 3.1. Optimizing Personalized Approaches to Intervention Strategies

Effective management of ADHD increasingly depends on tailoring interventions to optimize them for each patient’s unique needs. Traditional behavioral therapies have been widely applied, especially cognitive–behavioral therapy (CBT) [71,72,73]. CBT helps patients identify and modify maladaptive cognitive–behavioral patterns through structured therapist-guided feedback, improving executive function and emotional regulation [61,74,75]. In the intervention process, clinicians develop targeted treatment plans based on the individual’s age and the severity of symptoms. For example, treatment for adolescents with ADHD (ages 12–18) focuses on issues related to comorbid anxiety or emotional dysregulation [76]. In adults with ADHD, cognitive–behavioral therapy (CBT) enhances occupational efficiency and social adaptation. For those with severe inattention or comorbid depression, extended treatment cycles may be necessary [77,78,79]. Physical exercise and meditation have also shown the potential to reduce hyperactivity, improve emotional regulation, and enhance attention in children and adolescents with ADHD. Research indicates that physical exercise not only mitigates anxiety and depression but also enhances emotional regulation, executive function, and motor abilities in children with ADHD [80,81]. A systematic review emphasized that mindfulness-based interventions are especially effective in decreasing hyperactivity and impulsivity in children, while their impacts on attention and emotional regulation may differ depending on age [82]. These validated traditional intervention approaches demonstrate enduring effectiveness through their robust theoretical foundations and extensive clinical applications. They have not only been substantiated by numerous research studies but have also accumulated substantial empirical evidence and practical experience through widespread implementation.

Advanced neurotechnologies like neurofeedback and repetitive transcranial magnetic stimulation (rTMS) illustrate enhanced feedback mechanisms. Neurofeedback helps patients learn to self-regulate their brain activity using EEG feedback [64,83]. Therapists can adjust the treatment protocols based on regular evaluations of neural and behavioral data. This method allows researchers and clinicians to identify specific patterns and developmental trajectories of ADHD, which helps in creating more tailored interventions [84,85]. rTMS, which targets the right dorsolateral prefrontal cortex, has been shown to improve inhibitory control in adults with severe ADHD [86,87]. However, current protocols rely on clinician assessments rather than automated systems. Safety evaluations are also crucial for patients with comorbid epilepsy or anxiety [88,89].

Virtual reality (VR) and artificial intelligence (AI) have the potential to improve ADHD interventions by combining different types of data. These technologies offer new ways for clinicians to evaluate interventions. Immersive VR environments can replicate real-world situations, such as classrooms or social settings, to help with task engagement and social skills improvement. For instance, VR-based social skills training has been effective in enhancing social interactions among children with ADHD. This highlights its potential as a complement to traditional therapies like CBT [90]. Combining VR with data from eye tracking and EEG has shown promise in predicting ADHD symptoms and customizing interventions, especially for adults [91]. These methods may improve diagnostic accuracy and reveal clinically meaningful ADHD subtypes, which can guide personalized treatment plans. Additionally, AI-driven platforms could analyze complex datasets to suggest preliminary adjustments, which clinicians can then confirm through clinical observations before applying [92,93].

Future ADHD interventions should combine evidence-based traditional methods with technological tools under clinician supervision to manage complex cases. Building on established combinations like cognitive–behavioral therapy (CBT) with mindfulness or neurofeedback [66,94], future integration emphasizes clinician-guided technological augmentation. For instance, when using CBT alongside VR-based social training, therapists can analyze interaction data from simulated scenarios. This helps refine real-world skill-building strategies and improves task engagement in children with ADHD more effectively than traditional training [95]. Similarly, clinicians using gamified digital platforms can utilize structured feedback to boost treatment adherence in adolescents while monitoring attention and behavioral control [96]. These integrations are especially valuable for patients with comorbidities like anxiety or learning disorders. Clinicians can collect various types of data during treatment, including neural activity and behavioral patterns. By analyzing this information, they can create personalized treatment plans tailored to each patient’s specific needs. They can also adjust the next steps in the intervention based on feedback from multiple data sources at different stages of treatment [92,93].

The core of continuously optimizing intervention methods for more personalized treatment is clinician-centered solutions. These solutions are needed to address implementation challenges and ensure that all patients have fair access to treatment. While advanced tools like VR systems require costly hardware and technical expertise [97], therapists in resource-limited settings can use simpler alternatives, such as tablet-based cognitive training apps or mobile neurofeedback. These alternatives maintain core therapeutic principles through clinician-mediated feedback loops [98,99]. It is also important to develop tools that support clinician decision-making in different socioeconomic contexts. This includes creating interoperable data interfaces that combine school behavioral reports with clinical assessments. Collaborative frameworks should involve clinicians, developers, and policymakers to standardize review protocols for AI-generated recommendations and VR performance metrics. This approach ensures that technology enhances rather than replaces therapeutic expertise.

### 3.2. Systematic Feedback Strategies for ADHD Management

#### 3.2.1. Feedback Loops Shaped by Early Environments

Molecular genetic studies show that susceptibility to ADHD results from interactions between many genetic variations and epigenetic regulation. However, there are significant limitations in using genetic testing or epigenetic markers as clinical screening tools. Current technological constraints have two main aspects. First, the polygenic architecture of ADHD involves hundreds of minor-effect loci with low cumulative explanatory power, resulting in limited predictive validity of single tests [44]. Second, the changes in epigenetic markers over time create significant differences in methylation profiles between peripheral tissues and the central nervous system, making clinical application difficult [100]. Additionally, the high costs of advanced sequencing and bioinformatics analysis create economic burdens that limit public health scalability.

Active environmental intervention strategies can avoid the uncertainties of passive biomarker detection. These strategies can effectively prevent ADHD by eliminating risk factors, leading to a reduction in its incidence within the population. The environmental plasticity of epigenetic mechanisms provides a practical approach for ADHD prevention. By modifying environmental factors during pregnancy and early postpartum, we can target harmful epigenetic programming [37,39]. Maternal dietary patterns during pregnancy can influence long-term ADHD symptom risks in children. For example, omega-3 polyunsaturated fatty acid supplementation is linked to a lower occurrence of symptoms [101]. Cohort studies also reveal that higher maternal prenatal fiber intake is associated with lower ADHD symptom scores in offspring across different age groups [102]. Furthermore, animal research with mice further shows an important finding. A positive nurturing environment, such as frequent maternal licking, lowers cortisol levels in the offspring. This results in reduced stress levels in the young mice [103]. These findings provide valuable insights into how early environmental factors shape neurodevelopment. Optimizing epigenetic programming during these critical periods may improve neurodevelopmental outcomes and enhance adaptability to the environment.

However, for families where one or both parents have ADHD, implementing these environmental interventions can be particularly challenging [104]. Parents with ADHD often struggle to maintain consistent routines and manage stress. This can make it harder for them to effectively control environmental factors. As a result, they may make less healthy dietary choices, have greater exposure to substances like nicotine and alcohol, and experience higher psychological stress during pregnancy and early postpartum [105,106]. These challenges highlight the need for practical and accessible support systems that meet the specific needs of families affected by ADHD. Such systems can ensure that preventive strategies are both effective and feasible [106]. Therefore, advancing these strategies is crucial and requires attention from the whole society.

#### 3.2.2. Family and School Environment Feedback

Family and school environments are dynamic contexts where behavioral, emotional, and cognitive processes interact with external influences. These settings create opportunities for targeted interventions that can adapt to the evolving needs of individuals.

Within the family environment, positive parenting practices, consistent routines, and supportive communication can enhance self-regulation and executive functioning in children with ADHD. In contrast, high levels of family conflict or inconsistent parenting can worsen symptoms and create negative feedback loops that hinder intervention effectiveness [107,108]. To break such maladaptive cycles, interventions focused on parent training and family-based therapies have demonstrated significant benefits [109,110]. These approaches aim to disrupt harmful parenting patterns and the negative feedback they create. They establish positive feedback mechanisms within the family, fostering a nurturing environment that reinforces adaptive behaviors and reduces symptom severity. Moreover, these approaches can also support parental mental health [106,107]. This is critical because caregiver stress and emotional dysregulation can indirectly influence ADHD symptom trajectories. Additionally, parental mental health issues may stem from the parents themselves having ADHD, which can complicate their ability to provide effective support [107]. Improving parental resilience through stress management and emotional support can create a virtuous cycle of mutual support and behavioral improvement. This amplifies the overall impact of ADHD management strategies [106,108]. Thus, family-based interventions are important not only for the child but also for the entire family system.

The school environment is another critical area that has a significant impact on ADHD. Classroom settings, peer interactions, and teacher–student relationships shape a child’s social and academic experiences. These experiences, in turn, affect their behavioral and emotional development [111,112]. Effective school-based strategies focus on creating structured and supportive environments that meet the unique needs of children with ADHD [113,114]. For example, individualized education plans (IEPs) and classroom accommodations, such as preferential seating, extended time for tasks, and visual aids, can help reduce attentional and executive functioning challenges [115,116]. Teachers who are trained in ADHD-specific strategies can enhance these efforts by providing consistent feedback, encouraging positive reinforcement, and using behavioral management techniques tailored to each student [113].

Moreover, collaboration between families and schools is crucial for creating a unified feedback system that improves the effectiveness of ADHD interventions [117]. This system can include regular parent–teacher meetings, shared progress monitoring, and the involvement of professionals like school psychologists, counselors, or special education specialists. Such coordination helps align goals and strategies across home and school settings. This integrated feedback loop can improve immediate behavioral and academic outcomes while supporting the child’s long-term social adaptation and emotional resilience. The dynamic nature of these feedback mechanisms allows interventions to be continuously refined, tackling the unique challenges of ADHD while remaining adaptable over time.

#### 3.2.3. Social and Cultural Feedback

Social adaptation is a key focus in managing ADHD. Difficulties in building relationships, achieving academic success, and functioning in the workplace are strongly linked to negative long-term psychosocial outcomes [9,10]. In addition to family and school support, addressing these challenges requires a feedback-driven approach that continuously refines interventions based on cultural and contextual factors. Programs like the Summer Treatment Program have shown that evidence-based interventions can improve skills in children with ADHD. These interventions include a reward system, response cost point system, time-outs, and clear rules and routines. They can effectively enhance conflict resolution and social cue interpretation [118,119]. However, the effectiveness of these programs varies by cultural context, as the understanding and acceptance of ADHD can differ significantly across societies [120,121].

In societies where people are more aware of neurodevelopmental disorders, professional help is often easier to access. This allows families, teachers, and healthcare providers to communicate and work together more effectively [122]. However, the stigma surrounding mental health issues and the financial burden of treatment can hinder this communication process, preventing families from seeking timely care [123,124]. Additionally, in collectivist cultures, ADHD symptoms are sometimes misinterpreted as poor discipline or a lack of effort rather than as neurobiological factors. This misinterpretation can delay intervention and limit opportunities for feedback to inform culturally relevant care. For example, a cross-cultural study found that parents in Hong Kong and the UK have different thresholds for recognizing ADHD symptoms. This difference reflects broader cultural pressures, such as the emphasis on academic achievement and compliance in Hong Kong, which may lead to higher parental stress and stricter interpretations of children’s behavior [120]. Socioeconomic disparities further complicate the process of ADHD interventions. ADHD prevalence tends to be higher in low-income populations compared to high-income groups [125]. Addressing these disparities requires continuous input from community stakeholders to refine and scale interventions. For example, in low-resource settings, community-based programs that train parents and teachers in evidence-based behavioral techniques, such as behavioral parent training and school interventions, create cost-effective and scalable feedback mechanisms. These programs can improve parenting practices and reduce ADHD symptoms [126,127]. In high-resource settings, children often have access to more comprehensive diagnostic evaluations and multidisciplinary support. However, barriers such as treatment costs, limited insurance coverage, and stigma can disrupt the support system, especially for marginalized communities [128]. This highlights the need for policy reforms and outreach efforts that incorporate feedback from underserved populations to ensure equitable ADHD care.

Cultural adaptation is crucial for creating effective feedback mechanisms. Interventions that incorporate regional therapeutic customs and align with cultural norms are more likely to be sustainable [129]. For instance, adapting standardized role-playing into communal storytelling or traditional physical activities can enhance engagement among families and educators. This engagement provides valuable input to refine interventions [130]. Currently, cultural adaptation in ADHD interventions is underdeveloped. While ADHD is a neurodevelopmental disorder with universal diagnostic criteria, cultural differences can significantly influence how symptoms are perceived, reported, and managed. Insights from behavioral health interventions suggest that a systematic approach, including information gathering, preliminary design, testing, refinement, and final trials, can guide the development of culturally tailored ADHD interventions [131]. Culturally adapted interventions could address the stigma around ADHD in certain communities. They could also incorporate culturally relevant parenting practices or modify behavioral strategies to align with local norms. Future research should prioritize evaluating the effectiveness of these culturally specific adaptations, comparing them to standard interventions, and exploring how cultural factors, such as family dynamics and community support, influence treatment engagement and outcomes.

Long-term success in managing ADHD depends on integrating these culturally adapted strategies with systemic support to create continuous improvement processes. Positive reinforcement from families, schools, and community networks, along with campaigns to address stigma, can help children build resilience and foster social belonging. By emphasizing cultural relevance, resource efficiency, and collaborative implementation, feedback from diverse socioeconomic and cultural contexts can refine and scale interventions. This approach can ultimately lead to better outcomes for children with ADHD.

### 3.3. Dynamic and Personalized ADHD Care Strategies

Dynamic and personalized care strategies are crucial to ensure that ADHD interventions remain flexible, responsive, and sustainable over time. This approach emphasizes continuous monitoring, iterative refinement, and the integration of feedback from biological, behavioral, environmental, and social domains. It addresses the evolving nature of ADHD symptoms and the diverse challenges faced at different developmental stages, as conceptually shown in Figure 1.

In this dynamic feedback mechanism, data for diagnosing and assessing ADHD can be collected from multiple sources, such as neurophysiological markers, behavioral observations, and contextual inputs from family and school environments [64,83]. Technologies like smartwatches can monitor heart rate variability and stress levels, providing practical tools for tracking symptom progression [99]. Regular assessments of attention, impulsivity, and emotional regulation guide intervention adjustments. By integrating these feedback streams, clinicians can develop a comprehensive understanding of individual needs and make timely modifications to treatment plans.

For children, personalized strategies should focus more on foundational aspects such as nutrition and exposure to environmental toxins, which significantly impact neurodevelopment. Adequate intake of nutrients like omega-3 fatty acids and minimizing exposure to toxins are essential components of early intervention strategies [101,132]. Additionally, play-based therapies and parental training programs are vital for promoting self-regulation and executive functioning [118,126]. Notably, nutrition and physical exercise are critically important during childhood and throughout all developmental stages. In fact, these factors play a fundamental role in children’s neurodevelopment, emotional regulation, cognitive performance, and overall well-being [63,133]. During childhood, proper nutrition and regular physical activity are especially crucial for supporting brain development, enhancing cognitive functions, and establishing healthy lifestyle patterns [101].

Iterative refinement of interventions is essential to ensure care strategies remain aligned with the individual’s changing needs. ADHD symptoms often vary across life stages, requiring tailored approaches. Adolescents may need interventions focusing on academic performance, social skills, and emotional resilience. In contrast, adults often require support in managing workplace challenges and addressing comorbid conditions such as anxiety or depression. To effectively meet these diverse needs, it is important to combine various therapeutic modalities, such as cognitive–behavioral therapy (CBT), neurofeedback, and mindfulness-based interventions. This combination enhances the adaptability of treatment plans [66,94]. Additionally, advanced tools like AI-driven analytics and virtual reality environments can support this process by allowing for real-time adjustments. These technologies help predict symptom trajectories and optimize treatment strategies, ensuring that interventions remain responsive to the individual’s evolving challenges [95].

Building robust support systems across family, school, and social domains is equally critical for sustaining the effectiveness of personalized care strategies. Positive parenting practices, consistent routines, and open communication within families foster a nurturing environment that reinforces adaptive behaviors [109,110]. In schools, individualized education plans (IEPs), teacher training, and classroom accommodations help address the unique needs of children with ADHD [115,116]. Beyond the immediate family and school settings, broader social support networks, including community programs and peer groups, play a critical role in promoting long-term social adaptation and resilience, and they should be recognized as well [122].

Clinical professionals are central to these strategies, ensuring that interventions are individualized and effective [134]. ADHD symptoms vary widely among individuals and are often influenced by comorbid conditions, biological factors, and life circumstances. Clinicians must be central to decision-making, continuously re-evaluating and refining treatment plans to address these complexities. Their role is especially crucial in managing the interplay between ADHD and co-occurring conditions, such as anxiety, depression, or learning disabilities, which require personalized and targeted measures. It is the clinical professionals who are central to these strategies, ensuring that interventions are individualized and effective [134].

## 4. Insights into the Multidimensional Dynamic Model

### 4.1. Strengths and Limitations

The proposed *Multidimensional Dynamic Feedback Model* (MDFM) has significant strengths due to its comprehensive integration of biological, psychological, social, and environmental factors. This framework emphasizes the importance of early developmental periods. It recognizes that interventions during prenatal, early childhood, and adolescent stages can fundamentally shape long-term outcomes. Unlike reductionist approaches that focus on isolated aspects of ADHD pathology [30,31,32], this model accounts for the interactive nature of ADHD. It shows how early epigenetic programming and neurophysiological processes interact with family, school environments, and cultural contexts to influence developmental trajectories. The model’s dynamic feedback mechanisms are particularly valuable during critical developmental periods. Targeted interventions can effectively modify neural plasticity, behavioral patterns, and social adaptability through systematic environmental regulation and positive reinforcement loops [135]. By emphasizing early intervention and continuous monitoring throughout development, the model provides a strong foundation for individualized and adaptive interventions. These interventions can significantly change the course of ADHD manifestation and management. By prioritizing early identification and sustained support during crucial developmental stages, the model offers a comprehensive approach to optimize outcomes across the lifespan. It establishes a foundation for continued adaptation and success from childhood through adolescence and into adulthood.

Despite these advantages, there are limitations to consider. First, while incorporating novel technologies such as artificial intelligence (AI), virtual reality (VR), and wearable devices enhances the precision of ADHD interventions, it also raises ethical and legal concerns [92,136]. AI-driven analytics require large amounts of sensitive patient data, which raises issues of data privacy, consent, and potential misuse. Similarly, VR interventions, if not supervised by trained personnel, may cause psychological distress or worsen symptoms. To mitigate these risks, it is crucial to establish regulatory guidelines and standards for data handling. This involves using strong data encryption methods and ensuring that informed consent processes are clear and thorough. We should also develop protocols to anonymize data and protect patient identities. Furthermore, training programs for clinicians and researchers on ethical issues in technology use can help ensure that interventions respect patient dignity and confidentiality. Involving patients and their families in the development of these technologies can build trust and make sure their rights and preferences are respected. Another limitation is the real-world feasibility of implementing a highly integrated, technology-assisted approach [92]. The infrastructure, training, and collaboration needed to operationalize this model can be costly and complex. Resource-limited regions or smaller clinics without specialized staff and consistent funding may struggle to adopt these innovations, potentially widening the care gap. Therefore, a systematic rollout strategy that scales interventions based on local resources and includes flexible, lower-cost alternatives is essential.

### 4.2. Challenges and Future Directions

One practical challenge moving forward is refining this complex, dynamic framework and ensuring its acceptance among families, schools, healthcare systems, and policymakers. This will require time and effort. Current research shows promise for dynamic, feedback-adaptive strategies, but more studies are needed to confirm their effectiveness across various demographic, cultural, and socioeconomic contexts. To fully realize the model, it is essential to conduct longitudinal studies that track genetic, neuroimaging, and behavioral data from early development through adolescence and into adulthood. Such studies would help clarify how adaptive interventions influence neurodevelopmental trajectories over the lifespan and strengthen the model’s evidence base.

Broad social collaboration is also crucial. A key element is developing well-trained, multidisciplinary teams of professionals worldwide. While emerging technologies offer sophisticated tools for refining diagnoses and tailoring interventions, the complexity of ADHD means that decision-making and treatment coordination must remain with clinicians and mental health specialists. Within the dynamic feedback model, these professionals must combine biological and behavioral data with contextual information, including cultural factors, socioeconomic conditions, and family settings. Establishing cross-disciplinary training programs, regular workshops, and collaborations between institutions will help ensure that clinicians can integrate new technologies ethically and effectively.

Another important direction for the future is to scale simpler, cost-effective tools. This includes mobile neurofeedback systems, AI-based symptom monitoring apps, and brief VR interventions that can be used in lower-resource settings [68,137,138]. Policy reforms, along with engagement from stakeholders such as schools, parent advocacy groups, and healthcare providers, should aim to align these innovations with high-level oversight. This includes developing protocols for AI systems to collect and analyze patient data under established ethical standards while ensuring that VR environments are safe and culturally sensitive.

Interventions must also be adapted to reflect the cultural and socioeconomic variability in ADHD presentation and care-seeking behaviors [121,125]. Tailoring strategies for communities with limited mental health awareness or high stigma requires culturally relevant outreach, improved education about ADHD, and capacity-building for primary care or community health workers. Addressing comorbid conditions, such as anxiety, depression, and learning disabilities, is also critical [55,139]. These conditions often complicate ADHD challenges and reduce the effectiveness of standard treatments.

Ultimately, by advancing the understanding of ADHD as a dynamic system and creating interventions that align with continuous feedback across biological, psychological, and social levels, the proposed model offers a pathway to better prevent and manage ADHD throughout life. Through rigorous validation in longitudinal studies, user-friendly technologies, and a professional workforce that can ethically and creatively integrate multiple approaches, this multidimensional framework has the potential to improve ADHD care globally. This advancement could significantly enhance the quality of life for children and adolescents while also supporting the growing number of adults managing ADHD.
